# Inhibition of SIRT6 potentiates the anti-tumor effect of doxorubicin through suppression of the DNA damage repair pathway in osteosarcoma

**DOI:** 10.1186/s13046-020-01759-9

**Published:** 2020-11-17

**Authors:** Zhongkai Zhang, Sang Hoon Ha, Young Jae Moon, Usama Khamis Hussein, Yiping Song, Kyoung Min Kim, See-Hyoung Park, Ho Sung Park, Byung-Hyun Park, Ae-Ri Ahn, Sang-A Lee, Su Jin Ahn, Jung Ryul Kim, Kyu Yun Jang

**Affiliations:** 1 Department of Orthopedic Surgery, Jeonbuk National University Medical School, Jeonju, Republic of Korea; 2 Division of Biotechnology, Jeonbuk National University, Iksan, Republic of Korea; 3 Department of Biochemistry and Molecular Biology, Jeonbuk National University Medical School, Jeonju, Republic of Korea; 4 Research Institute of Clinical Medicine of Jeonbuk National University-Biomedical Research Institute of Jeonbuk National University Hospital and Research Institute for Endocrine Sciences, Jeonju, Republic of Korea; 5 Department of Pathology, Jeonbuk National University Medical School, Jeonju, Republic of Korea; 6 Faculty of Science, Beni-Suef University, Beni-Suef, Egypt; 7 Department of Bio and Chemical Engineering, Hongik University, Sejong, Republic of Korea

**Keywords:** Osteosarcoma, SIRT6, doxorubicin, apoptosis, DNA damage

## Abstract

**Background:**

SIRT6 has diverse roles in cells, and the role of SIRT6 in tumorigenesis is controversial. Considering the role of SIRT6 as an inducer of DNA damage repair, it might be involved in resistance to anti-cancer therapy.

**Methods:**

We evaluated the prognostic significance of SIRT6 in 37 osteosarcomas and investigated the therapeutic efficacy of SIRT6 on the anticancer effects of doxorubicin, olaparib, and ATM inhibitor.

**Results:**

Immunohistochemical expression of SIRT6 was significantly associated with shorter overall survival and relapse-free survival of osteosarcoma patients, especially in patients who received adjuvant chemotherapy. In U2OS and KHOS/NP osteosarcoma cells, knock-down of SIRT6 significantly potentiated apoptotic effects of doxorubicin and SIRT6 overexpression induced resistance to doxorubicin. Moreover, SIRT6 induced the DNA damage repair pathway and SIRT6-mediated resistance to doxorubicin was attenuated by blocking the DNA damage repair pathway with olaparib and ATM inhibitor.

**Conclusions:**

This study suggests that suppression of SIRT6 in combination with doxorubicin might be an effective modality in the treatment of osteosarcoma patients, especially for osteosarcomas with shorter survival with high expression of SIRT6.

## Background

SIRT6 is a member of the sirtuin family and important in the regulation of cellular physiology and tumorigenesis [1–5]. SIRT6 is best known for its roles in the regulation of cellular proliferation, energy metabolism, aging, and DNA damage repair [1–3, 5–8]. Especially, the functions of SIRT6 in the repair of DNA damage raise the possibility that SIRT6 may be involved in tumorigenesis [9]. However, there are controversial reports on the role of SIRT6 in tumorigenesis and tumor progression. Especially, the effect of SIRT6 in tumor growth has been reported controversially according to the type of cancer [4, 10–13]. Despite the roles of SIRT6 as a sensor to repair DNA damage, higher expression of SIRT6 is related to increased proliferation and invasiveness of cancer cells [14–16]. Furthermore, elevated expression of SIRT6 was associated with shorter survival of cancer patients in the breast, stomach, lung, ovary, and lymphoma [14, 16–19]. In contrast, loss of SIRT6 is a factor associated with shorter survival of breast and liver cancer patients in other reports [13, 20]. Even with the same MDA-MB-231 breast cancer cells, there are conflicting reports of SIRT6 either increasing or decreasing the proliferation of these cells [13, 14]. These controversial reports on the role of SIRT6 in cancer growth suggest that the effect of SIRT6 expression might differ according to the condition of the cancer itself or the cancer-associated environment.

One of the critical factors determining the survival of cancer patients is the responsiveness of cancer to anti-cancer therapy. Regarding resistance to anti-cancer therapy of cancer cells, various factors such as activation of the DNA damage repair pathway, immune evasion, metabolic change, and the stemness of cancer cells have important roles [21, 22]. Especially, the DNA damage repair pathway is tumor suppressive in normal cells, but it induces resistance of cancer cells to genotoxic anti-cancer therapies [23, 24]. Based on these paradoxical roles of the molecules of the DNA damage repair pathway, inhibitors of this pathway, such as Poly (ADP-ribose) polymerase (PARP) inhibitors, have been developed as anti-cancer agents [11, 21]. Therefore, when based on the role of SIRT6 in the DNA damage repair pathway [1, 8, 25], SIRT6 might be an inducer of therapeutic resistance [26].

Osteosarcoma is the most common primary bone malignancy [27]. Despite recent advances in the understanding of human cancers, osteosarcomas are often refractory to treatment [28]. Recently, there are increasing reports focusing on the roles of the sirtuins, especially on SIRT6, in human cancers [4, 29, 30]. Furthermore, the DNA damage repair pathway is a promising target of cancer treatment [11]. In osteosarcoma cells, the PARP inhibitor, olaparib, potentiates the therapeutic efficacy of doxorubicin [31]. However, there have been limited studies on the roles of SIRT6 in osteosarcoma [32–35]. Furthermore, the role of SIRT6 in the progression of osteosarcoma has been reported controversially [32, 33, 35]. However, when considering the role of SIRT6 in the repair of DNA damage, SIRT6 might be involved in the effectiveness of anti-cancer treatment [2, 8, 25, 26, 29]. Therefore, this study evaluated the role and effect of SIRT6 expression on osteosarcoma by investigating the expression of SIRT6 in human osteosarcomas and assessing the role of SIRT6 in resistance to the treatment of doxorubicin in conjunction with the role of SIRT6 on the repair of DNA damage.

## Methods

### Osteosarcoma patients

In this study, 37 osteosarcomas of bone that underwent surgical resection at the Jeonbuk National University Hospital between January 1998 and December 2012 were evaluated. The cases with a complete medical record, histologic slides, and paraffin-embedded tissue blocks were included in this study. All cases were reviewed according to the World Health Organization classification of bone tumors [27] and the American Joint Committee on Cancer staging system [36]. The clinicopathologic factors of osteosarcoma evaluated in this study were age (< 30 y *versus* ≥ 30 y), sex (male *versus* female), tumor size (≤ 8 cm *versus* > 8 cm), tumor stage (I *versus* II-III), histologic grade (low *versus* high), lymph node metastasis (absence *versus* presence), distant metastasis at diagnosis (absence *versus* presence), and latent distant metastasis (absence *versus* presence). Latent distant metastasis is defined as a relapse of osteosarcoma at a distant organ during follow-up after operation. The clinical information was obtained by reviewing the medical records. There were no patients who received preoperative chemotherapy, but twenty-six patients received adjuvant chemotherapy. This study was performed with approval of the institutional review board approval at Jeonbuk National University Hospital, and the requirement for informed consent was waived (IRB number, CUH 2018-08-040-001).

### Immunohistochemical staining and scoring in the tissue microarray of human osteosarcoma

Immunohistochemical staining for SIRT6 was performed with tissue microarray (TMA) sections for osteosarcoma. The TMA cores were established to contain primarily tumor cells in the core without any degenerative change. Two 3.0 mm cores were established for each case. The TMA sections were deparaffinized and antigen retrieval was performed by boiling with a microwave oven for 20 minutes in pH 6.0 antigen retrieval buffer (DAKO, Glostrup, Denmark). The tissue sections were incubated with anti-SIRT6 (1:100, Cell Signaling Technology, Beverly, MA) antibodies. The slides immunostained for SIRT6 were evaluated by two pathologists (KYJ and KMK) under a multi-viewing microscope and the scoring performed with consensus without information for the case. The immunohistochemical expression of SIRT6 was scored by adding the staining intensity score and staining area score. The staining intensity was scored from zero to three (0; no expression, 1; weak expression, 2; intermediate expression, and 3; strong expression), and the staining area was scored from zero to five (0: no stained cells; 1: 1%, 2: 2 ~ 10%, 3: 11 ~ 33%; 4: 34 ~ 66%; and 5: 67 ~ 100%) [10, 31, 37–39]. Thereafter, the final immunohistochemical staining score was obtained by adding the scores obtained from each TMA core, which ranged from zero to sixteen [38, 39].

### Cell culture, chemicals, and transfection

This study used two human osteosarcoma cell lines, U2OS (Korean Cell Line Bank, Seoul, Korea) and KHOS/NP. KHOS/NP cells were kindly provided by Chang-Bae Kong (Department of Orthopedic Surgery, Korea Institute of Radiological and Medical Science). The cells were cultured in Dulbecco’s modified Eagle’s medium (Gibco BRL, Gaithersburg, MD) containing 10% FBS (Gibco BRL) and 1% penicillin/streptomycin (100 U/mL) at 37 °C in a humidified incubator under 5% CO_2_. This study used doxorubicin (D1515, Sigma, St. Louis, MO), KU-55,933, an ATM inhibitor, (SML1109, Sigma), and olaparib, a PARP inhibitor (SC-302,017, Santa Cruz Biotechnology, Santa Cruz, CA). The vector for SIRT6-specific shRNA was purchased from GenePharma Co. (GenePharma, Shanghai, China). The SIRT6 duplex had the sense and antisense sequences 5′-CACCGCTACGTTGACGAGGTCATGATTCAAGAGATCATGACCTCGTCAACGTAGCTTTTTTG-3′ and 5′-GATCCAAAAAAGCTACGTTGACGAGGTCATGATCTCTTGAATCATGACCTCGTCAACGTAGC-3′, respectively [16]. A pFLAG-CMV-2 plasmid vector was used as a control vector. The vector overexpressing wild-type SIRT6 (pFLAG2_SIRT6) was synthesized by Cosmogenetech Co. Ltd. (Cosmogenetech Co. Ltd, Seoul, Korea) [16]. Transfection was performed using Lipofectamine 3000 (Invitrogen, Carlsbad, CA).

### Cell proliferation assay and colony-forming assay

The proliferation of cells was evaluated with the Cell Proliferation ELISA, BrdU (colorimetric) assay (Roche, Mannheim, Germany) and colony-forming assay. The BrdU assay was performed with seeding of U2OS (0.8 × 10^3^) and KHOS/NP (0.8 × 10^3^) cells on 96-well plates and measured with Bio-Rad model 680 microtiter plate reader (Bio-Rad, Hercules, CA) at a wavelength of 370 nm. The colony-forming assay was performed by plating U2OS cells (2 × 10^3^) and KHOS/NP cells (2 × 10^3^) in 24-well culture plates. The culture plates were stained with 0.01% crystal violet (Sigma) seven days after seeding. The number of colonies was quantified by using the Colony Area ImageJ Plugin (https://imagej.nih.gov/ij).

### Western blot analysis and immunoprecipitation

The cells were lysed with Mammalian Protein Extraction Reagent (Thermo Fisher Scientific, Rockford, lL) containing 1 × phosphatase inhibitor cocktails 2, 3 (Sigma). For immunoprecipitation, 500 µg of protein precleared with Mammalian Protein Extraction Reagent was incubated with antibodies, as indicated, overnight and then with protein G-agarose (Thermo Fisher Scientific) for two hours. Cell lysates were separated by 10% SDS-PAGE and transferred to PVDF membranes. The blot was probed with primary antibodies. The primary antibodies used for western bolt were SIRT6 (Cell Signaling Technology), cleaved PARP1 (Cell Signaling Technology), cleaved caspase 3 (Cell Signaling Technology), BCL2 (Santa Cruz Biotechnology), BAX (Santa Cruz Biotechnology), Chk2 (Thr68) (Cell Signaling Technology), phosphorylated Chk2 (p-Chk2, Cell Signaling Technology), ATM (Cell Signaling Technology), phosphorylated ATM (Ser1981) (p-ATM, Cell Signaling Technology), P53 (Santa Cruz Biotechnology), phosphorylated P53 (Ser15) (p-P53,Santa Cruz Biotechnology), H2AX (Cell Signaling Technology), γH2AX (Cell Signaling Technology), IgG (Cell Signaling Technology), and GAPDH (Santa Cruz Biotechnology). The bands of western blot were quantified using an ImageJ software (https://imagej.nih.gov/ij).

### Flow cytometry analysis for apoptosis

Apoptosis was assessed *via* flow cytometry analysis with staining for FITC-conjugated annexin V and propidium iodide using the apoptosis detection kit (Invitrogen). The suspended cells in 100 µL of binding buffer at a concentration of 1 × 10^6^ cell/mL were incubated with 5 µL of annexin V-FITC and 5 µL of propidium iodide for 15 min at room temperature in the dark. The samples were analyzed using a BD FACSCalibur system (Becton-Dickinson, San Jose, CA).

### Orthotopic tumor model

In the orthotopic xenograft model, six-week-old male BALB/c nude mice (Orient Bio, Gyeonggi-Do, Korea) were used. The tumor in the right proximal tibia was induced by injecting 2.5 × 10^6^ KHOS/NP cells transfected with empty vectors, shRNA for SIRT6, or plasmid for wild-type SIRT6 into the marrow space under anesthesia. Four mice were used in each group. Two weeks after tumor cell inoculation, mice were injected intraperitoneally with doxorubicin (4 mg/kg in dimethyl sulfoxide) once a week. The body weight and tumor volume were measured every five days. The tumor volumes were calculated as “length x width x height x 0.52”. The animals were euthanized with sodium pentobarbital at 20 days after treatment with doxorubicin. Histologic sections of the resected tumors were stained with hematoxylin and eosin and immunohistochemical staining for SIRT6 and Ki67. The tissue sections from the tumor were incubated with anti-SIRT6 (Cell Signaling Technology, Beverly, MA) and anti-Ki67 (Clone MIB-1, DAKO, Glostrup, Denmark) antibodies. Immunohistochemical staining for Ki67 was quantified as the percent of tumor cells with nuclear expression of Ki67. The amount of apoptosis in resected tumors was evaluated with a Terminal deoxynucleotidyl transferase dUTP nick end labeling (TUNEL) assay using the DeadEnd colorimetric TUNEL system (Promega, Madison, MA). To quantify TUNEL-positive cells, ten microscopic images were obtained in each tumor with a 40x objective lens (Plan Apo40/0.95NA, Nikon, Japan) and a digital camera (Nikon DXm1200F, Nikon, Japan). The area of one microscopic image was 0.086 mm^2^. Therefore, 0.86 mm^2^ per tumor was evaluated to count the number of TUNEL-positive cells. Animal experiments were approved by the institutional animal care and use committee of Jeonbuk National University (approval number: CBNU 2018 − 109).

### Statistical analysis

Immunohistochemical positivity for SIRT6 was determined by receiver operating characteristic curve analysis. The cut-off point was determined at the point with the highest area under the curve to predict the death of osteosarcoma patients [15, 20, 28]. The prognosis of osteosarcoma patients was evaluated for overall survival (OS) and relapse-free survival (RFS). The endpoint of follow-up was June 2014. An event in OS analysis was the death of the patient from osteosarcoma and the duration was calculated from the date of operation to the date of death or last follow-up. An event in RFS analysis was a relapse of any type and death from osteosarcoma and the duration was calculated from the date of operation to the date of the event or last follow-up. Statistical analysis for survival was performed by univariate and multivariate Cox proportional hazards regression analyses and Kaplan-Meier survival analysis. The significance between the evaluated factors was evaluated with the Pearson’s χ2 test and the Student’s *t*-test. All experiments were performed in triplicate, and representative data are presented. SPSS software (IBM, version 20.0, CA) was used throughout, and *P* values less than 0.05 were considered statistically significant.

## Results

### The expression of SIRT6 is associated with poor prognosis of osteosarcoma patients

**Fig. 1:**
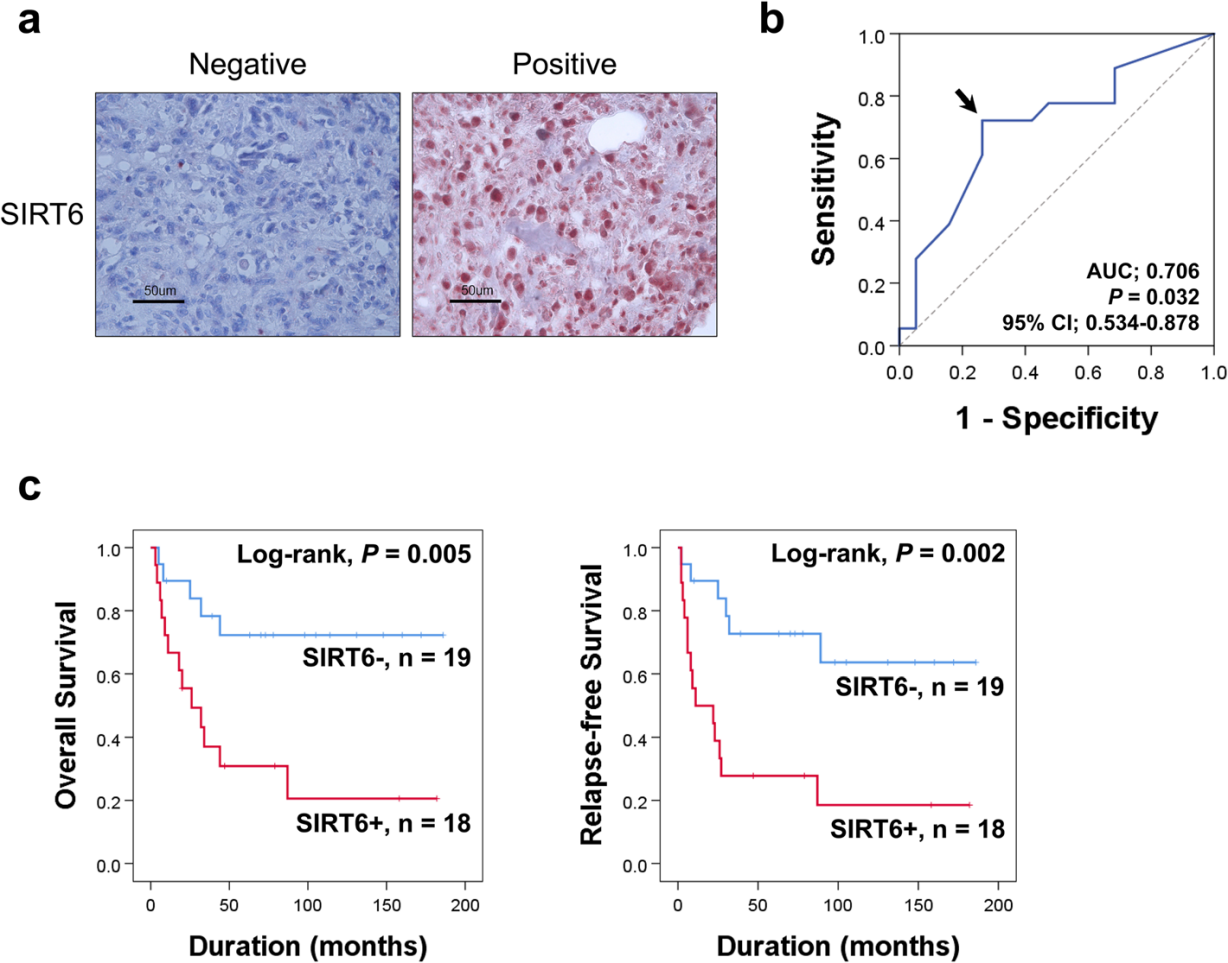
Statistical and survival analysis according to the immunohistochemical expression of SIRT6 in osteosarcoma. **a** Immunohistochemical expression of SIRT6 in human osteosarcoma tissue. **b** Receiver operating characteristic curve analysis to determine a cut-off point of immunohistochemical expression of SIRT6. The cut-off point was determined as the point with the highest area under the curve (AUC), indicated by the arrow. **c** Kaplan-Meier survival analysis according to SIRT6 expression for overall survival and relapse-free survival in 37 osteosarcoma patients

Immunohistochemical expression of SIRT6 in human osteosarcoma is presented in Fig. 1a. The cut-off point of the immunohistochemical staining score for SIRT6 expression was determined at the point with the highest area under the curve, which was nine (Fig. 1b). The cases with immunohistochemical staining scores equal to, or greater than, nine were considered SIRT6-positive. With this cut-off point, SIRT6 positivity was significantly associated with latent distant metastasis of osteosarcoma patients (*P* = 0.005) (Table 1). However, there was no significant association between SIRT6 expression and tumor stage or histologic grade (Table 1).

**Table 1:** The clinical characteristics of 27 osteosarcomas and their correlation with the immunohistochemical expression of SIRT6

Characteristics		No.	SIRT6	
			Positive	*P*
Age, years	< 30	24	11 (46%)	0.642
	≥ 30	13	7 (54%)	
Sex	Male	25	13 (52%)	0.556
	Female	12	5 (42%)	
Tumor size	≤ 8 cm	19	9 (47%)	0.873
	> 8 cm	18	9 (50%)	
Stage	I	11	4 (36%)	0.331
	II, III, & IV	26	14 (54%)	
Histologic grade	1 & 2	10	3 (30%)	0.167
	3 & 4	27	15 (56%)	
Lymph node metastasis	Absence	34	17 (50%)	0.580
	Presence	3	1 (33%)	
Distant metastasis at diagnosis	Absence	30	14 (47%)	0.618
	Presence	7	4 (57%)	
Latent distant metastasis	Absence	28	10 (36%)	0.005
	Presence	9	8 (89%)	

In univariable analyses, age, tumor size, stage, histologic grade, lymph node metastasis, distant metastasis, and SIRT6 expression were significantly associated with OS or RFS (Table 2). Especially, SIRT6-positivity indicated a 4.012 fold greater risk of death of osteosarcoma patients (95% CI, 1.419–11.344; *P* = 0.009) and had a 4.151 fold greater risk of relapse of tumor or death of patients (95% CI, 1.570-10.971; *P* = 0.004) (Table 2). Kaplan-Meier survival analysis also indicated that SIRT6 expression is significantly associated with OS (Log-rank, *P* = 0.005) and RFS (Log-rank, *P* = 0.002) (Fig. 1c). Multivariable analyses were performed with standard variables significantly associated with OS or RFS in univariable analyses. Age, tumor size, stage, lymph node metastasis, distant metastasis, histologic grade, and SIRT6 expression were included in multivariable analyses. Multivariable analyses showed tumor size (OS; *P* = 0.023, RFS; *P* = 0.018), distant metastasis (OS; *P* = 0.008, RFS; *P* = 0.050), and SIRT6 expression (OS; *P* = 0.001, RFS; *P* < 0.001) as independent prognostic indicators of osteosarcoma patients (Table 3). SIRT6-positivity had a 6.797 fold greater risk of death of osteosarcoma patients (95% CI, 2.129–21.702) and had a 6.516 fold greater risk of relapse of tumor or death of patients (95% CI, 2.234–19.001) (Table 3).

**Table 2:** Univariate Cox proportional hazards regression analysis for the overall survival and relapse-free survival of osteosarcoma patients

Characteristics	No.	OS		RFS	
		HR (95% CI)	*P*	HR (95% CI)	*P*
Age, years, ≥ 30 (vs. <30)	13/37	2.398 (0.938–6.134)	0.068	2.925 (1.210–7.070)	0.017
Sex, male (vs. female)	25/37	1.140 (0.402–3.229)	0.806	1.513 (0.541–4.228)	0.430
Tumor size, > 8 cm (vs. ≤ 8 cm)	18/37	3.975 (1.401–11.280)	0.010	3.067 (1.204–7.813)	0.019
Stage, ≥ II (vs.. I)	26/37	4.829 (1.107–21.058)	0.036	3.267 (0.953–11.206)	0.060
Histologic grade, 3 & 4 (vs. 1 & 2)	27/37	4.502 (1.031–19.666)	0.045	5.130 (1.184–22.222)	0.029
Lymph node metastasis, presence (vs.. absence)	3/37	5.373 (1.026–28.150)	0.047	2.981 (0.628–14.142)	0.169
Distant metastasis, presence (vs. absence)	7/37	5.347 (1.793–15.950)	0.003	3.802 (1.295–11.163)	0.015
SIRT6, positive (vs. negative)	18/37	4.012 (1.419–11.344)	0.009	4.151 (1.570-10.971)	0.004

*OS* overall survival; *RFS* relapse-free survival; *HR* hazard ratio; *95% CI* 95% confidence interval

**Table 3:** Multivariate survival analysis in overall osteosarcoma patients

Characteristics	OS			RFS	
	HR (95% CI)	*P*		HR (95% CI)	*P*
Tumor size, > 8 cm (vs. ≤ 8 cm)	3.795 (1.205–11.953)	0.023		3.680 (1.249–10.842)	0.018
Distant metastasis, presence (vs. absence)	6.200 (1.603–23.984)	0.008		3.450 (0.997–11.934)	0.050
SIRT6, positive (vs. negative)	6.797 (2.129–21.702)	0.001		6.516 (2.234–19.001)	< 0.001

*OS* overall survival; *RFS* relapse-free survival; *HR* hazard ratio; *95% CI* 95% confidence interval. The variables included in multivariate analysis were age, tumor size, stage, lymph node metastasis, distant metastasis, histologic grade, and SIRT6 expression

**Fig. 2:**
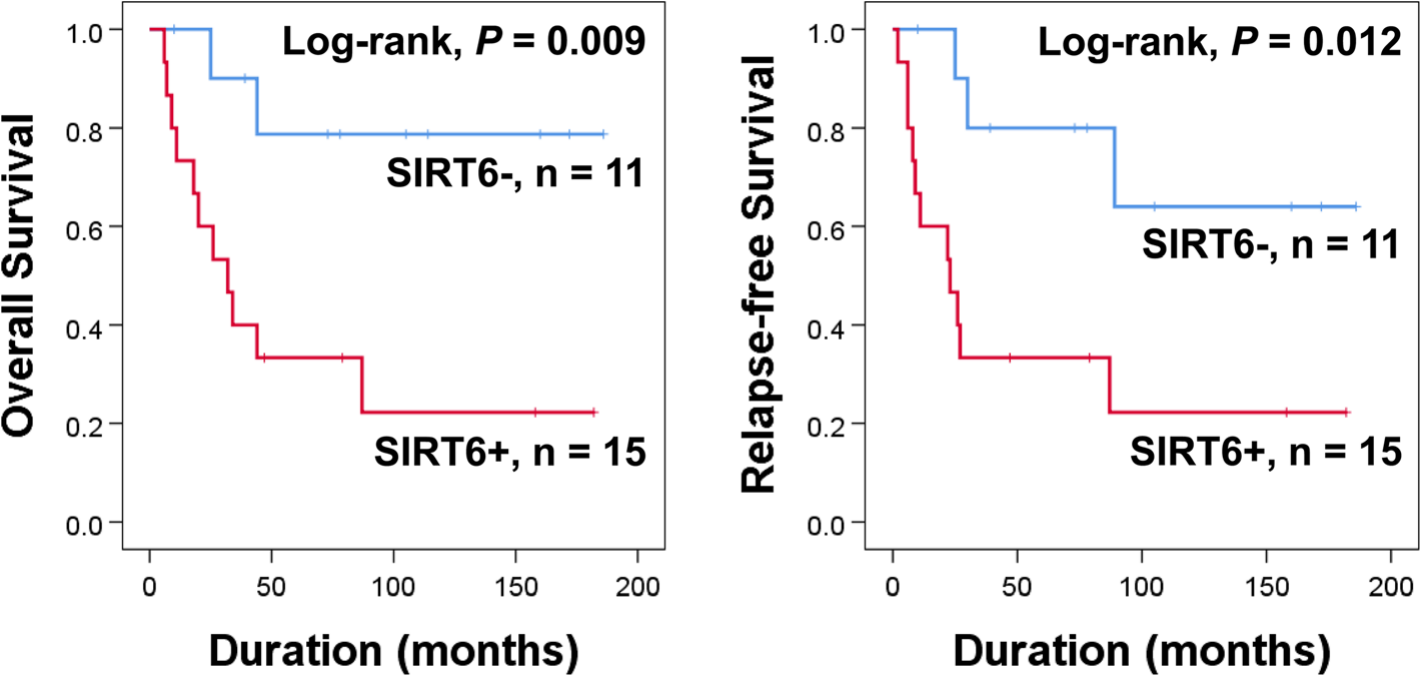
Kaplan-Meier survival analysis according to SIRT6 expression for overall survival and relapse-free survival in 26 osteosarcoma patients who performed adjuvant chemotherapy

Furthermore, we performed additional survival analysis with 26 osteosarcoma patients who were treated with postoperative adjuvant chemotherapy. In this sub-group of osteosarcoma patients, SIRT6 expression was an independent indicator of poor prognosis of osteosarcoma patients with univariable and multivariable analyses. SIRT6 expression predicted a 6.988 fold greater risk of death of osteosarcoma patients (95% CI, 1.439–33.930; *P* = 0.016) and had a 6.930 fold greater risk of relapse of tumor or death of patients (95% CI, 1.727–27.814; *P* = 0.006) (Table 4). Kaplan-Meier survival curves for the expression of SIRT6 on OS (Log-rank, *P* = 0.009) and RFS (Log-rank, *P* = 0.012) are presented in Fig. 2.

**Table 4:** Univariate and multivariate survival analysis in 26 osteosarcoma patients who treated with adjuvant chemotherapy

Characteristics	No.	OS			RFS	
		HR (95% CI)	*P*		HR (95% CI)	*P*
Univariate analysis						
SIRT6, positive (vs. negative)	15/26	5.855 (1.290-26.563)	0.022		4.523 (1.245–16.434)	0.022
Multivariate analysis						
Tumor size, > 8 cm (vs. ≤ 8 cm)					3.560 (1.055–12.009)	0.041
Distant metastasis, presence (vs. absence)		48.868 (2.759-865.448)	0.008		10.649 (1.186–95.614)	0.035
SIRT6, positive (vs. negative)		6.988 (1.439–33.930)	0.016		6.930 (1.727–27.814)	0.006

*OS* overall survival; *RFS* relapse-free survival; *HR* hazard ratio; *95% CI* 95% confidence interval. The variables included in multivariate analysis were age, tumor size, stage, lymph node metastasis, distant metastasis, histologic grade, and SIRT6 expression

### Suppression of SIRT6 potentiates the effect of doxorubicin on the proliferation of osteosarcoma cells

**Fig. 3:**
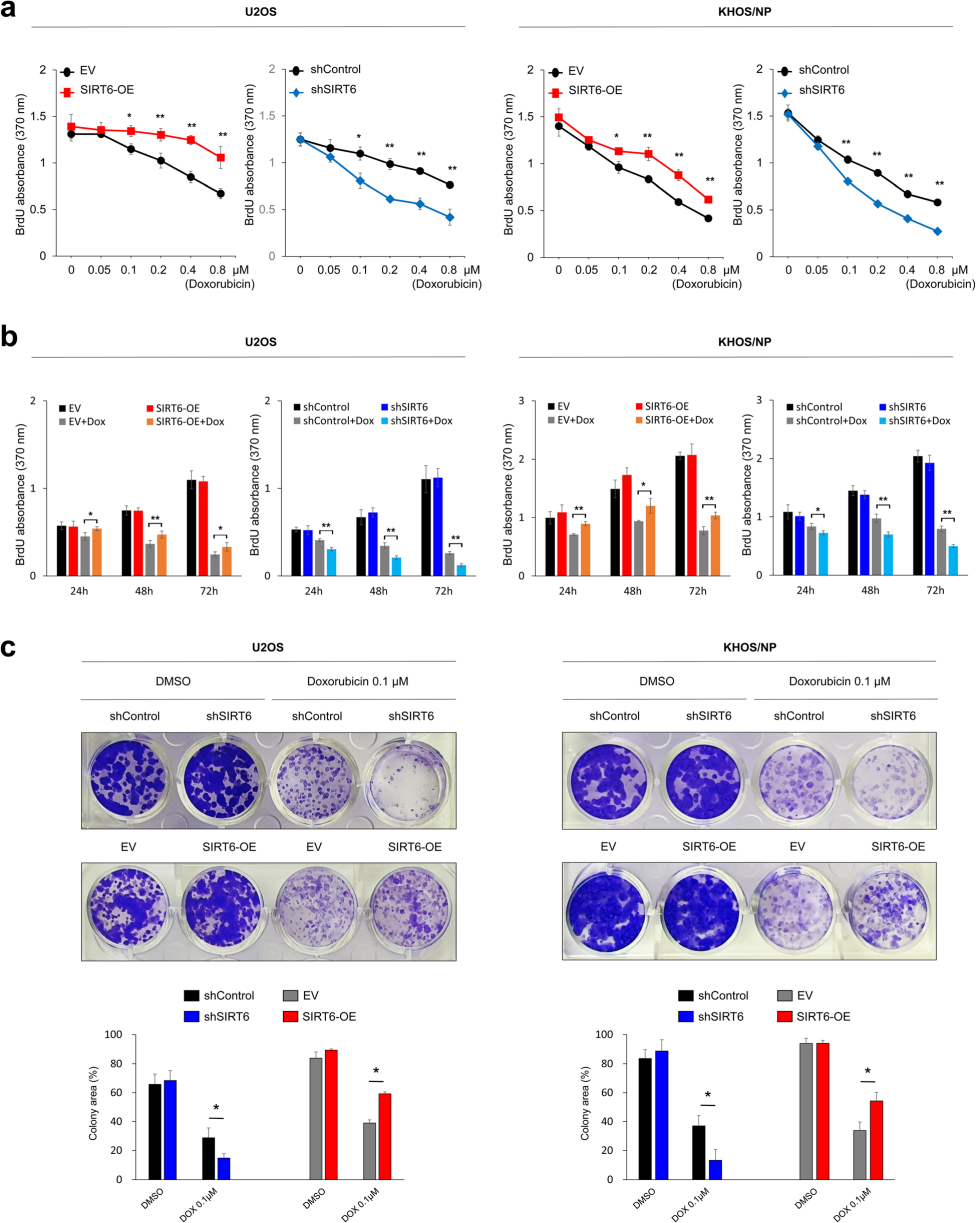
Suppression of SIRT6 synergizes doxorubicin-mediated inhibition of proliferation of osteosarcoma cells. **a** U2OS and KHOS/NP osteosarcoma cells were transfected with control shRNA, shRNA for SIRT6, empty vector, or wild-type SIRT6 and treated with the indicated dose of doxorubicin (0, 0.05, 0.1, 0.2, 0.4, or 0.8 µM) for 24 hours, and then measured with a BrdU proliferation assay. **b** The U2OS and KHOS/NP cells transfected with control shRNA, shRNA for SIRT6, empty vector, or wild type-SIRT6 were treated with 0.1 µM doxorubicin and measured after 24, 48, and 72 hours for proliferation with a BrdU assay. **c** A colony-forming assay was performed by seeding U2OS cells (2 × 10^3^) and KHOS/NP cells (2 × 10^3^) transfected with control shRNA, shRNA for SIRT6, empty vector, or wild type-SIRT6, and treating DMSO or 0.1 µM doxorubicin in 24-well culture plates. *; *P* < 0.05, **; *P* < 0.001

Based on the prognostic significance of SIRT6 expression in osteosarcoma patients, especially in the patients who received adjuvant chemotherapy, we evaluated the effects of SIRT6 expression on the anti-tumor activity of doxorubicin in osteosarcoma cells. As shown in Fig. 3a and b, when we did not treat with doxorubicin (at a dose of zero for doxorubicin in Fig. 3a and b), the knock-down or overexpression of SIRT6 did not influence the proliferation of U2OS and KHOS/NP osteosarcoma cells. At a dose of 0.05 µM doxorubicin, the knock-down or overexpression of SIRT6 did not influence on the proliferation of U2OS and KHOS/NP osteosarcoma cells (Fig. 3a). However, overexpression of SIRT6 significantly increased the proliferation of cells compared with control cells, and knock-down of SIRT6 significantly decreased the proliferation of cells compared with control cells with treatment of doxorubicin from 0.1 to 0.8 µM (Fig. 3a). When we treated 0.1 µM doxorubicin for 24, 48, and 72 hours, overexpression of SIRT6 significantly increased the proliferation of cells compared with control cells, and knock-down of SIRT6 significantly decreased the proliferation of cells compared with control cells with treatment of doxorubicin from 0.1 to 0.8 µM (Fig. 3b and c). Therefore, overexpression of SIRT6 attenuated the anti-proliferative effect of doxorubicin and knock-down of SIRT6 potentiated the effect of doxorubicin at concentrations of doxorubicin at or above 0.1 µM (Fig. 3).

**Fig. 4:**
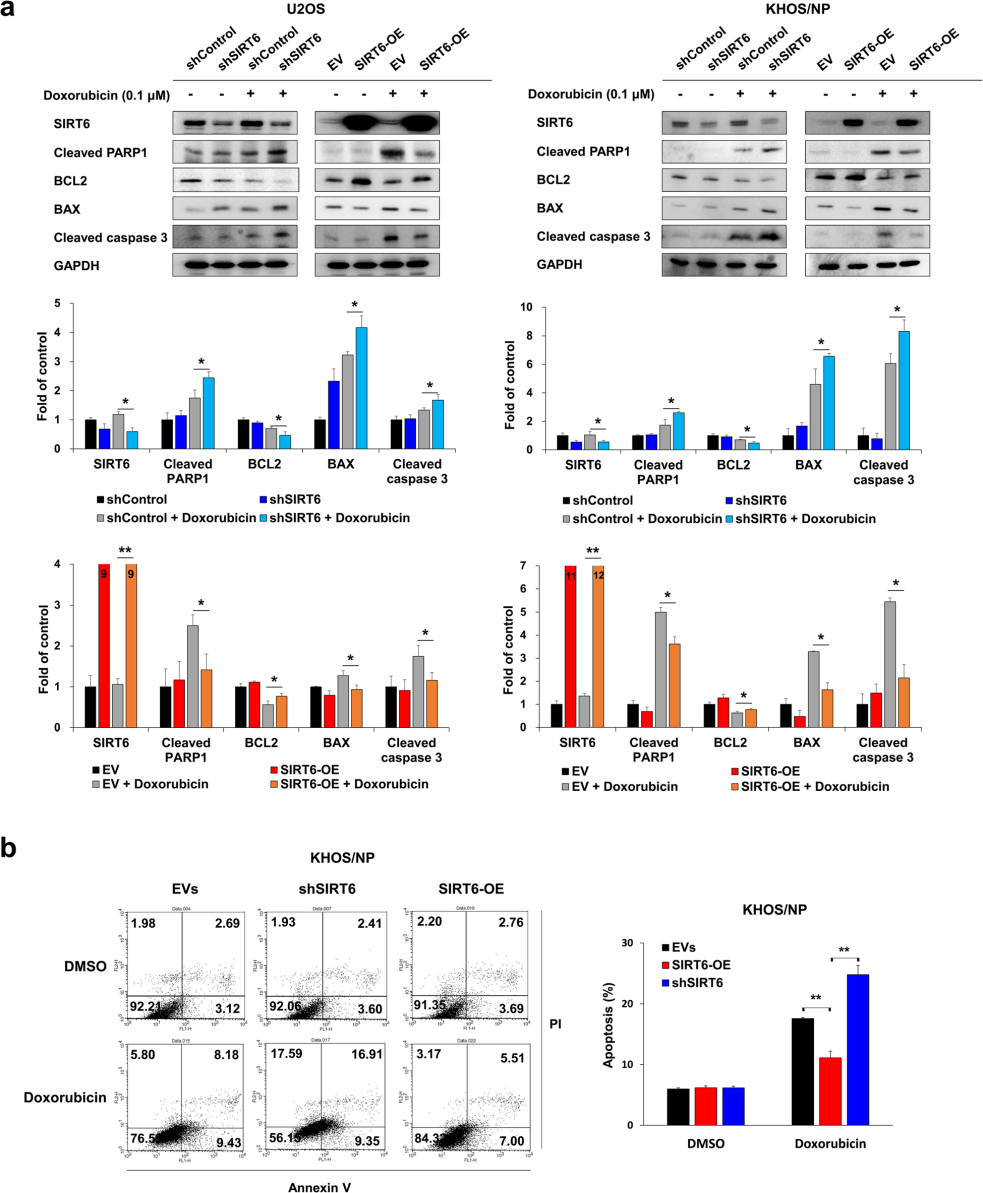
Suppression of SIRT6 synergistically increases doxorubicin-mediated apoptosis of osteosarcoma cells. **a** U2OS and KHOS/NP osteosarcoma cells were transfected with control shRNA, shRNA for SIRT6, empty vector, or wild-type SIRT6 and treated with 0.1 µM doxorubicin. Western blots for SIRT6, cleaved PARP1, BCL2, BAX, cleaved caspase 3, and GAPDH were performed. Densitometry analysis of western blots was performed in triplicate and measured by using ImageJ software. **b** KHOS/NP cells transfected with empty vectors, shRNA for SIRT6, or wild-type SIRT6 were treated with DMSO or 0.1 µM doxorubicin for 24 hours and flow-cytometry analysis performed with staining for propidium iodide and Annexin V. *; *P* < 0.05, **; *P* < 0.001

**Fig. 5:**
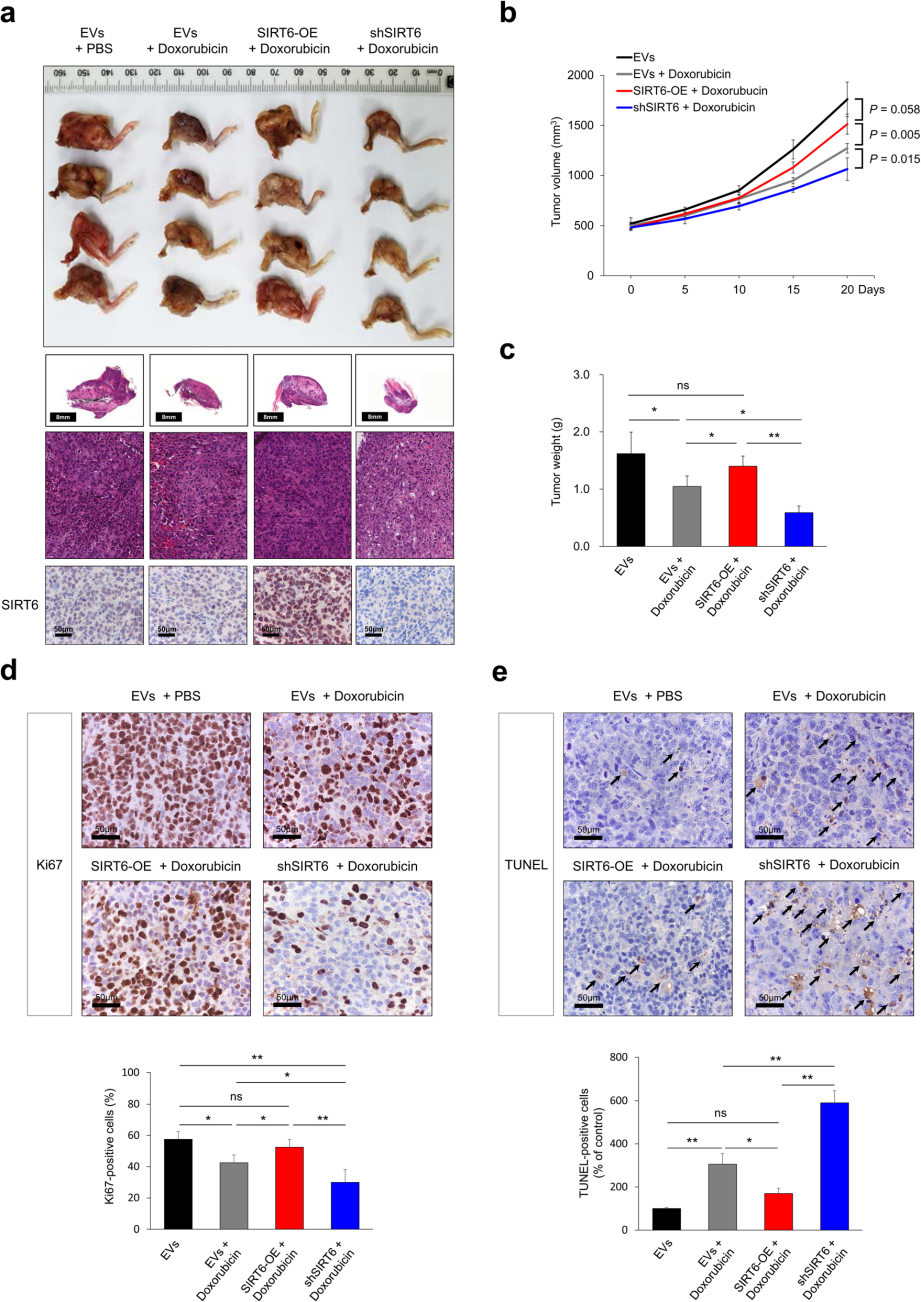
Suppression of SIRT6 potentiates doxorubicin-induced suppression of *in vivo* growth of osteosarcoma cells. **a** KHOS/NP cells were transfected with empty vectors, shRNA for SIRT6, or wild-type SIRT6, and 2.5 × 10^6^ KHOS/NP cells were injected into the marrow space of the right proximal tibia under anesthesia. Two weeks after tumor cell inoculation, doxorubicin (4 mg/kg in DMSO, once a week) was injected intraperitoneally. Macroscopic and microscopic images were acquired after euthanizing mice 20 days after the start of doxorubicin treatment. Immunohistochemical staining for SIRT6 was performed in resected tumors. **b** The tumor volume was measured every five days. The tumor volumes were calculated as “length x width x height x 0.52”. **c** Weight of tumors measured after resection. **d** Immunohistochemical staining for Ki67 was performed in resected tumors and the percentage of Ki67-positive cells was evaluated for each experimental group. **e** The number of TUNEL-positive cells was counted in ten microscopic images obtained at high-power magnification (x400 magnification) for each tumor. *; *P* < 0.05, **; *P* < 0.001

**Fig. 6:**
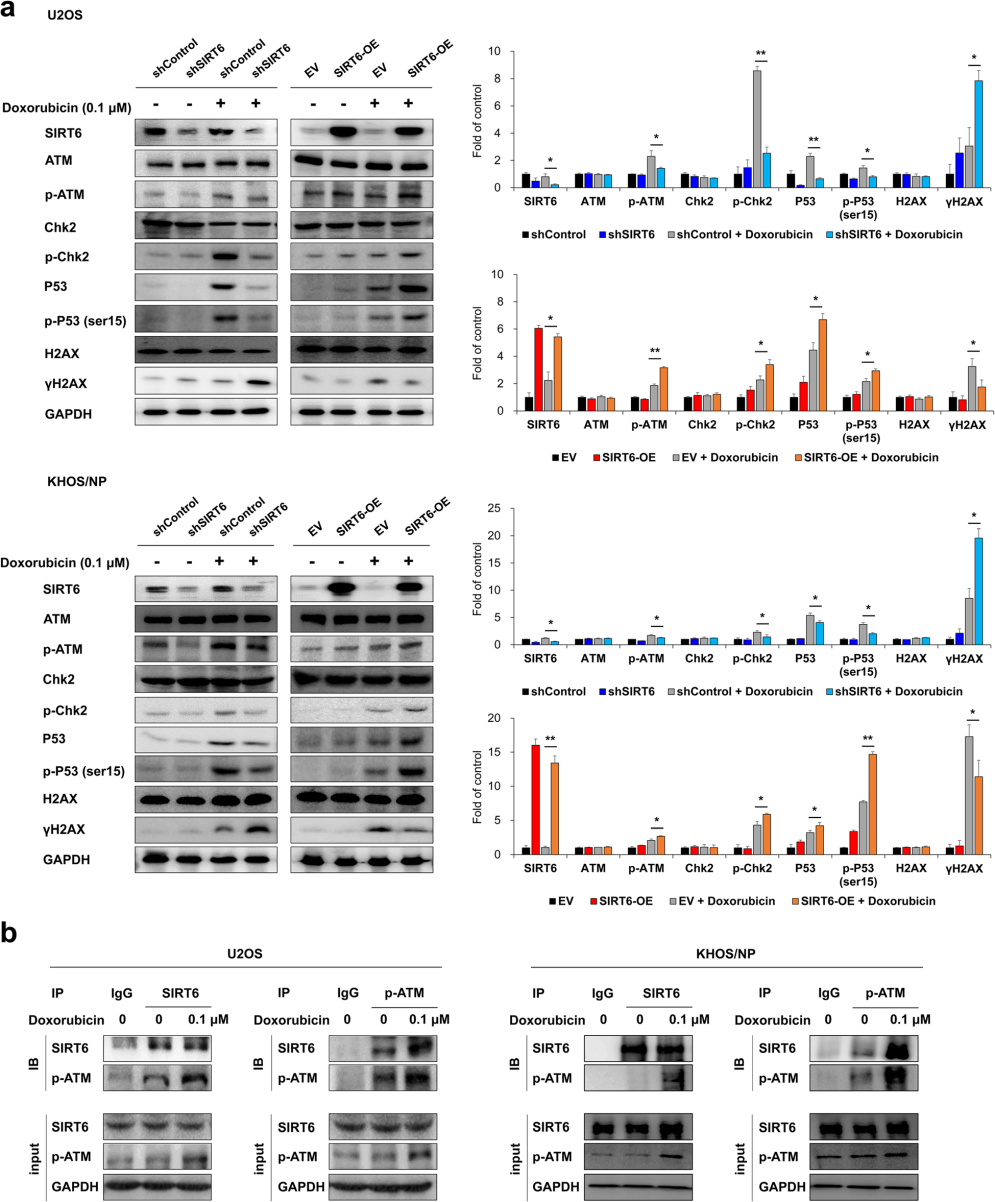
SIRT6 stimulates the DNA damage repair pathway in doxorubicin-induced DNA damage. **a** U2OS and KHOS/NP cells transfected with control shRNA, shRNA for SIRT6, empty vector, or wild type-SIRT6 were treated with DMSO or 0.1 µM doxorubicin, and western blotting performed for SIRT6, ATM, phosphorylated ATM (p-ATM), Chk-2, phosphorylated Chk2 (p-Chk2), P53, phosphorylated P53 [p-P53(ser15)], H2AX, γH2AX, and GAPDH. Densitometry analysis of western blots was performed in triplicate and measured by using ImageJ software. **b** Whole cell lysates of U2OS and KHOS/NP treated with DMSO (0, control) or 0.1 µM doxorubicin were subjected to immunoprecipitation (IP) with an anti-SIRT6 antibody, anti-p-ATM antibody, or an isotype IgG (control) followed by immunoblotting (IB) with antibodies for SIRT6 and p-ATM. Input bands were obtained by performing western blot for SIRT6, p-ATM, and GAPDH. *; *P* < 0.05, **; *P* < 0.001

**Fig. 7:**
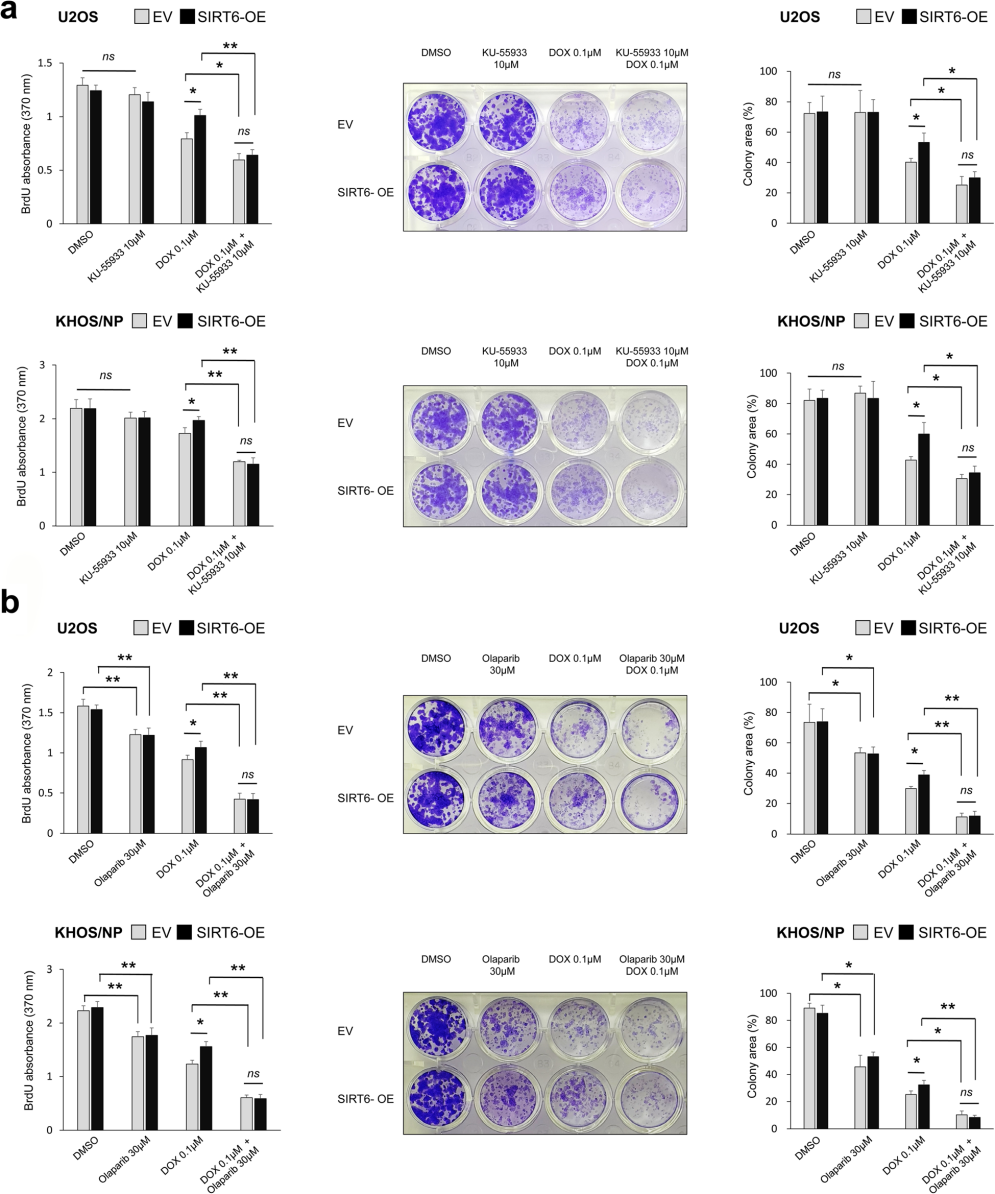
Co-treatment of ATM inhibitor KU-55,933 or the PARP inhibitor olaparib with doxorubicin synergistically inhibited the proliferation of SIRT6-overexpressing U2OS and KHOS/NP osteosarcoma cells. **a** U2OS and KHOS/NP cells transfected with empty vector or wild type-SIRT6 were treated with DMSO, 10 µM KU-55,933, 0.1 µM doxorubicin, or 0.1 µM doxorubicin and 10 µM KU-55,933. BrdU proliferation assay was performed by seeding of 0.8 × 10^3^ U2OS cells and 0.8 × 10^3^ KHOS/NP cells, and absorbance measured 24 hours after seeding. Colony-forming assay was performed by seeding U2OS cells (2 × 10^3^) and KHOS/NP cells (2 × 10^3^) in 24-well culture plates. **b** U2OS and KHOS/NP cells transfected with empty vector or wild type-SIRT6 were treated with DMSO, 30 µM olaparib, 0.1 µM doxorubicin, or 0.1 µM doxorubicin and 30 µM olaparib. The BrdU proliferation assay was performed by seeding of 0.8 × 10^3^ U2OS cells and 0.8 × 10^3^ KHOS/NP cells, and the absorbance measured 24 hours after seeding. Colony-forming assays were performed by seeding U2OS cells (2 × 10^3^) and KHOS/NP cells (2 × 10^3^) in 24-well culture plates. *; *P* < 0.05, **; *P* < 0.001

**Fig. 8:**
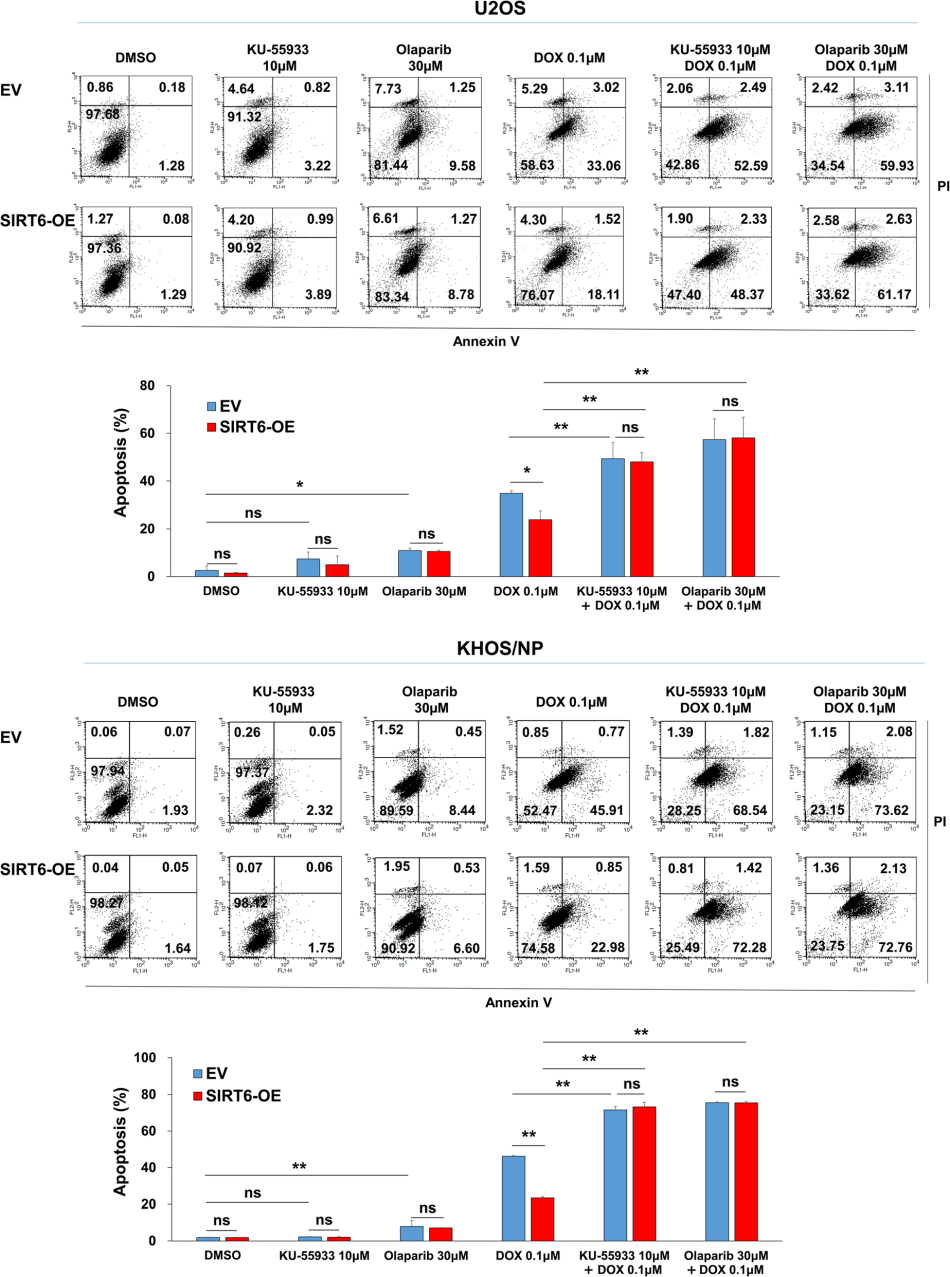
Co-treatment of ATM inhibitor KU-55,933 or the PARP inhibitor olaparib with doxorubicin synergistically increases apoptosis of SIRT6-overexpressing osteosarcoma cells. U2OS and KHOS/NP cells were transfected with empty vector or wild-type SIRT6 and treated with DMSO, 10 µM KU-55,933, 30 µM olaparib, 0.1 µM doxorubicin, 0.1 µM doxorubicin and 10 µM KU-55,933, or 0.1 µM doxorubicin and 30 µM olaparib for 24 hours and flow-cytometry analysis performed with staining for propidium iodide and Annexin V. *; *P* < 0.05, **; *P* < 0.001

### Suppression of SIRT6 potentiates the cytotoxic effect of doxorubicin by increasing the apoptosis of osteosarcoma cells

Based on the cytotoxic activity of doxorubicin, we further evaluated the effects of SIRT6 expression on apoptosis of osteosarcoma cells when co-treated with 0.1 µM doxorubicin. The effect of SIRT6 expression on apoptotic signaling was minimal without the treatment of doxorubicin (Fig. 4a). However, in the cells treated with doxorubicin, the knock-down of SIRT6 increased expressions of cleaved PARP1, BAX, and cleaved caspase 3, but decreased expression of BCL2; conversely, overexpression of SIRT6 decreased the expression of cleaved PARP1, BAX and cleaved caspase 3, but increased expression of BCL2 in both U2OS and KHOS/NP cells (Fig. 4a). Flow cytometric apoptotic analysis also showed more apoptosis of KHOS/NP cells with knock-down of SIRT6 and decreased apoptosis of KHOS/NP cells with overexpression of SIRT6 in a condition of co-treatment of 0.1 µM doxorubicin (Fig. 4b).

### SIRT6 influence on ***in vivo*** growth of osteosarcoma cells with treatment of doxorubicin

We further evaluated the expression of SIRT6 on *in vivo* tumor growth in mice treated with doxorubicin. The treatment of doxorubicin itself significantly decreased tumor growth. Furthermore, knock-down of SIRT6 significantly potentiated the inhibitory effect of doxorubicin on *in vivo* tumor growth (Fig. 5). The immunohistochemical expression of SIRT6 in *in vivo* tumors is presented in Fig. 5a. Compared to the group receiving only doxorubicin treatment, tumor growth was significantly decreased with knock-down of SIRT6 (Fig. 5a and b, and 5c). However, the effect of the anti-tumor effect of doxorubicin was attenuated by overexpression of SIRT6. There was no significant difference between the EVs group and SIRT6-overexpression with the doxorubicin treated group (Fig. 5a and b, and 5c). Immunohistochemical staining for Ki67 in the xenograft tumors showed more Ki67-positive cells in the SIRT6-overexpressing group compared with the group with induced knock-down of SIRT6 upon treatment with doxorubicin (Fig. 5d). TUNEL staining in the xenograft tumors showed that the number of TUNEL-positive cells increased with the treatment of doxorubicin. However, the doxorubicin-mediated increase in apoptosis was attenuated with overexpression of SIRT6 (Fig. 5e). There were no significant differences in the number of TUNEL-positive cells between the EVs group and the SIRT6-overexpressing doxorubicin treated group (Fig. 5e). However, knock-down of SIRT6 was associated with an increase in apoptosis upon treatment with doxorubicin. The number of TUNEL-positive apoptotic cells significantly increased with SIRT6 knock-down in the doxorubicin treated group compared with the group treated with doxorubicin only (Fig. 5e). There was no toxic effect or distant metastasis on internal organs in four experimental groups.

### Blocking of the DNA damage repair pathway with an ATM inhibitor and the PARP inhibitor, olaparib, potentiates the cytotoxic effects of doxorubicin in osteosarcoma cells overexpressing SIRT6

Recently, a role of SIRT6 in the DNA damage repair pathway has been reported [1, 8, 25]. Moreover, the effects of SIRT6 on the proliferation and apoptosis of osteosarcoma cells under the treatment of doxorubicin raise the possibility that the SIRT6-mediated DNA damage repair pathway might affect the cytotoxic effect of doxorubicin. Consistently, under treatment of doxorubicin, overexpression of SIRT6 increased expression of phosphorylated ATM, phosphorylated Chk2, P53, and phosphorylated P53, while knock-down of SIRT6, especially when combined with 0.1 µM doxorubicin treatment, decreased the expression of phosphorylated ATM, phosphorylated Chk2, P53, and phosphorylated P53 (Fig. 6a). In addition, the expression of γH2AX increased with the knock-down of SIRT6 and decreased with overexpression of SIRT6 under treatment of 0.1 µM doxorubicin (Fig. 6a). Furthermore, to validate the interaction between SIRT6 and p-ATM, we compared the levels of SIRT6 and p-ATM using co-immunoprecipitation and immunoblotting assays with cell lysates obtained after treatment with doxorubicin. As shown in Fig. 6b, the interaction between SIRT6 and p-ATM increased in response to doxorubicin-mediated DNA damage in both U2OS and KHOS/NP cells. These findings suggest that overexpression of SIRT6 induces resistance to the genotoxic agent doxorubicin by repairing damage to escape apoptosis. Therefore, we evaluated the effect of co-treatment of doxorubicin and an ATM inhibitor/PARP inhibitor in osteosarcoma cells in which the overexpression of SIRT6 had been induced. The treatment of ATM inhibitor KU-55,933 alone did not significantly inhibit the proliferation of U2OS and KHOS/NP cells (Fig. 7a). In contrast, the treatment of 30 µM olaparib alone significantly inhibited the proliferation of U2OS and KHOS/NP osteosarcoma cells (Fig. 7b). In addition, the cytotoxic effect of doxorubicin was attenuated with the overexpression of SIRT6 in U2OS and KHOS/NP osteosarcoma cells (Fig. 7a and b). Furthermore, co-treatment of KU-55,933 or the PARP inhibitor olaparib with doxorubicin synergistically inhibited the proliferation of SIRT6-overexpressing U2OS and KHOS/NP osteosarcoma cells (Fig. 7a and b). In addition, we performed flow cytometry analysis for apoptosis after co-treatment of doxorubicin and an ATM inhibitor/PARP inhibitor in osteosarcoma cells in which the overexpression of SIRT6 had been induced. Upon treatment with doxorubicin, overexpression of SIRT6 attenuates doxorubicin-mediated increase of apoptosis in U2OS and KHOS/NP osteosarcoma cells (Fig. 8). However, upon treatment of doxorubicin, SIRT6-overexpression mediated decrease of apoptosis after treatment of doxorubicin was reversed by co-treatment of KU-55,933 or the PARP inhibitor olaparib (Fig. 8). Furthermore, co-treatment of KU-55,933 or the PARP inhibitor olaparib with doxorubicin synergistically increased apoptosis of U2OS and KHOS/NP osteosarcoma cells (Fig. 8).

## Discussion

In this study, we demonstrate that high expression of SIRT6 is significantly associated with shorter survival of osteosarcoma patients as an independent indicator of shorter OS and RFS of osteosarcoma patients. In line with these results, the expression of SIRT6 was higher in osteosarcoma tissue compared with normal bone tissue [35]. Furthermore, high expression of SIRT6 was significantly associated with more metastasis and shorter OS and disease-free survival of osteosarcoma patients [35]. Consistently, the expression of SIRT6 was elevated in cancers compared with normal counterpart cells in the colon, esophagus, melanocytes, thyroid, and lymph node [12, 15, 19, 40, 41]. In addition, high expression of SIRT6 was associated with lymph node metastasis of colorectal cancers and thyroid papillary carcinomas [12, 15] and was associated with poor prognosis of breast cancer, ovary cancer, gastric cancer, lung cancer, and diffuse large B-cell lymphoma patients [14, 16–19]. However, controversially, higher expression of SIRT6 was associated with favorable prognosis of breast cancer patients [13], and loss of SIRT6 was associated with progression of hepatocellular carcinomas [20] and colon carcinomas [5]. These controversial reports on the role of SIRT6 in human cancers suggest that the impact of SIRT6 expression in cancer patients might differ depending on cancer type and/or characteristics of cancer patients included in the study [4, 14]. Therefore, further study is needed to clarify the various roles of SIRT6 in tumorigenesis and cancer progression.

The prognostic significance of SIRT6 expression might be related to the role of SIRT6 on the proliferation of cancer cells, and SIRT6 is known as an important regulator of cellular proliferation [13–16]. However, the impact of SIRT6 on the proliferation of cancer cells has been reported controversially [13–16]. Regarding a role for SIRT6 as an oncogenic molecule, SIRT6 stimulated the proliferation of cancer cells by stimulating the Wnt/β-catenin pathway [16]. In addition, SIRT6 stimulated the invasiveness of cancer cells by activating the epithelial-to-mesenchymal transition pathway in breast cancer, ovarian cancer, lung cancer, and osteosarcoma cells [14, 16, 35, 42]. Especially, increased invasiveness of SIRT6-overexpressing cancer cells was associated with activation of the ERK1/2-MMP9 pathway [35] and secretion of IL8 and TNF cytokines [43]. In non-small cell lung cancer, SIRT6 induced epithelial-to-mesenchymal transition by regulating the snail/KLF4 pathway [42]. However, controversially, elevated expression of SIRT6 inhibited the proliferation and invasiveness of osteosarcoma cells [32], and breast cancer cells [13]. Overexpression of SIRT6 inhibited the proliferation of HepG2 liver cancer cells by suppressing the ERK1/2 pathway [44]. In a model of idiopathic pulmonary fibrosis, SIRT6 inhibited epithelial-to-mesenchymal transition [45]. However, in this study, despite the prognostic significance of SIRT6 on the survival of osteosarcoma patients, the regulation of expression of SIRT6 did not significantly influence the proliferation of osteosarcoma cells. Therefore, further study focusing on the role of SIRT6 according to the specific type of cancer is needed.

In our results, despite the prognostic significance of SIRT6 expression, there was only one potential prognostic clinicopathological variable significantly associated with SIRT6 expression. A factor significantly associated with SIRT6 expression was latent distance metastasis of osteosarcoma. In addition, SIRT6-positivity was significantly associated with shorter OS and RFS in subgroups of osteosarcoma patients who received adjuvant chemotherapy. In addition, overexpression of SIRT6 induced resistance to doxorubicin-mediated apoptosis of osteosarcoma cells and knock-down of SIRT6 sensitized doxorubicin-mediated apoptosis of osteosarcoma cells. These results suggest that SIRT6-related differences in the prognosis of osteosarcoma patients might be related to SIRT6-mediated resistance to anti-cancer therapy. Supportively, knock-down of SIRT6 sensitized non-small cell lung cancer cells to paclitaxel treatment [46], and inhibition of SIRT6 sensitized lymphoma cells to doxorubicin [19]. Furthermore, regarding responsiveness of cancer cells on anti-cancer therapy, one mechanism of interest is the role of the molecules related with the DNA damage repair pathway in therapeutic resistance [2, 24]. As it has been reported in breast and ovarian cancers, mutation in BRCA1/2 is correlates with early development of cancer, which are responsive to genotoxic anti-cancer therapies [21, 23]. In addition, higher expression of molecules associated with the DNA damage repair pathways such as BRCA1/2, PARP, and γH2AX were associated with shorter survival of human cancer of the breast, ovary, and soft tissue [47–49]. Furthermore, based on this rationale, various PARP inhibitors are FDA-approved drugs with cancer patients with defects in BRCA1/2 such as ovary, breast, and prostatic cancer (OncoKB database, https://www.oncokb.org, accessed July 31, 2020). In osteosarcoma cells, defects in BRCA1/2 make tumors susceptible to the PARP inhibitor talazoparib [50]. Furthermore, there is an increasing number of reports indicated that SIRT6 mediates the DNA damage repair pathways as a DNA double-strand break sensor by activating PARP1 [2, 8, 25]. In this study, overexpression of SIRT6 induced the DNA damage repair pathway. Upon treatment with doxorubicin, the interaction between SIRT6 and p-ATM increased, and overexpression of SIRT6 increased expression of p-ATM and p-Chk2 and consequently decreased expression of γH2AX; conversely, knock-down of SIRT6 decreases the expression of p-ATM and p-Chk2. In addition, SIRT6 overexpression-mediated resistance to doxorubicin was attenuated with co-treatment of the ATM inhibitor KU-55,933 or the PARP inhibitor olaparib. Furthermore, treatment with KU-55,933 or olaparib synergizes with the anti-cancer effect of doxorubicin in both control osteosarcoma cells and osteosarcoma cells with induced overexpression of SIRT6. In line with our results, inhibition of PARP *via* knock-down of PARP1 or PARP inhibitor sensitized osteosarcoma cells and Ewing sarcoma cells to radiation or chemotherapeutic drugs [31, 51, 52]. In contrast, knock-down of SIRT6 increased viability of breast cancer cells treated with trastuzumab [13]. However, despite controversial reports on the role of SIRT6 as a therapeutic target of human cancer, our results indicate an important role of SIRT6 in the treatment of osteosarcoma in blocking of the SIRT6-mediated DNA repair pathway synergizes with the anti-cancer effect of the genotoxic anti-cancer agent doxorubicin. Furthermore, although a novel small molecular inhibitor of SIRT6, OSS_128167, suppressed *in vivo* growth of diffuse large B-cell lymphoma cells [19], the study of well-established therapeutic agents targeting SIRT6 have been limited [38], our result suggests that using of an agent targeting the DNA damage repair pathway such as a PARP inhibitor might be helpful for patients in the poor prognostic group of osteosarcoma patients which express high levels of SIRT6. In addition, when considering the reports that elevated expression of SIRT6 is associated with poor prognosis of human cancers, agents which block the DNA damage repair pathway might be useful for the treatment of other human cancer expressing high levels of SIRT6.

## Conclusions

In conclusion, our results indicate that SIRT6 expression might be useful as a novel prognostic indicator for osteosarcoma patients, especially for patients who received adjuvant chemotherapy. Furthermore, our results show that suppression of SIRT6 or inhibition of the SIRT6-mediated DNA damage repair pathway synergizes with the anti-cancer effect of doxorubicin. Therefore, a combination of inhibition of the SIRT6/SIRT6-mediated DNA damage repair pathway with conventional genotoxic anti-cancer therapies might be a new therapeutic stratagem for the poor prognostic group of osteosarcomas which have high expression of SIRT6.

## Data Availability

The datasets used and/or analyzed during the current study are available from the corresponding author upon reasonable request.

## Abbreviations

95% CI: 95% confidence interval
HR: Hazard ratio
OS: Overall survival
PARP: Poly (ADP-ribose) polymerase
RFS: Relapse-free survival
TMA: Tissue microarray

## References

[1] Lombard DB, Schwer B, Alt FW, Mostoslavsky R (2008). SIRT6 in DNA repair, metabolism and ageing. J Intern Med.

[2] Mao Z, Hine C, Tian X, Van Meter M, Au M, Vaidya A, Seluanov A, Gorbunova V (2011). SIRT6 promotes DNA repair under stress by activating PARP1. Science.

[3] Mostoslavsky R, Chua KF, Lombard DB, Pang WW, Fischer MR, Gellon L, Liu P, Mostoslavsky G, Franco S, Murphy MM (2006). Genomic instability and aging-like phenotype in the absence of mammalian SIRT6. Cell.

[4] de Ceu Teixeira M, Sanchez-Lopez E, Espina M, Garcia ML, Durazzo A, Lucarini M, Novellino E, Souto SB, Santini A, Souto EB (2019). Sirtuins and SIRT6 in Carcinogenesis and in Diet. Int J Mol Sci..

[5] Sebastian C, Zwaans BM, Silberman DM, Gymrek M, Goren A, Zhong L, Ram O, Truelove J, Guimaraes AR, Toiber D (2012). The histone deacetylase SIRT6 is a tumor suppressor that controls cancer metabolism. Cell.

[6] Kanfi Y, Naiman S, Amir G, Peshti V, Zinman G, Nahum L, Bar-Joseph Z, Cohen HY (2012). The sirtuin SIRT6 regulates lifespan in male mice. Nature.

[7] Etchegaray JP, Zhong L, Mostoslavsky R (2013). The histone deacetylase SIRT6: at the crossroads between epigenetics, metabolism and disease. Curr Top Med Chem.

[8] Onn L, Portillo M, Ilic S, Cleitman G, Stein D, Kaluski S, Shirat I, Slobodnik Z, Einav M, Erdel F, et al. SIRT6 is a DNA double-strand break sensor. Elife. 2020;9. 10.7554/eLife.51636 PMC7051178 31995034

[9] Cea M, Cagnetta A, Adamia S, Acharya C, Tai YT, Fulciniti M, Ohguchi H, Munshi A, Acharya P, Bhasin MK (2016). Evidence for a role of the histone deacetylase SIRT6 in DNA damage response of multiple myeloma cells. Blood.

[10] Allred D, Harvey JM, Berardo M, Clark GM (1998). Prognostic and predictive factors in breast cancer by immunohistochemical analysis. Mod Pathol.

[11] Oei AL, Vriend LE, van Leeuwen CM, Rodermond HM, Ten Cate R, Westermann AM, Stalpers LJ, Crezee J, Kanaar R, Kok HP (2017). Sensitizing thermochemotherapy with a PARP1-inhibitor. Oncotarget.

[12] Geng CH, Zhang CL, Zhang JY, Gao P, He M, Li YL (2018). Overexpression of Sirt6 is a novel biomarker of malignant human colon carcinoma. J Cell Biochem.

[13] Thirumurthi U, Shen J, Xia W, LaBaff AM, Wei Y, Li CW, Chang WC, Chen CH, Lin HK, Yu D (2014). MDM2-mediated degradation of SIRT6 phosphorylated by AKT1 promotes tumorigenesis and trastuzumab resistance in breast cancer. Sci Signal.

[14] Bae JS, Park SH, Jamiyandorj U, Kim KM, Noh SJ, Kim JR, Park HJ, Kwon KS, Jung SH, Park HS (2016). CK2alpha/CSNK2A1 Phosphorylates SIRT6 and Is Involved in the Progression of Breast Carcinoma and Predicts Shorter Survival of Diagnosed Patients. Am J Pathol.

[15] Qu N, Hu JQ, Liu L, Zhang TT, Sun GH, Shi RL, Ji QH (2017). SIRT6 is upregulated and associated with cancer aggressiveness in papillary thyroid cancer via BRAF/ERK/Mcl1 pathway. Int J Oncol.

[16] Bae JS, Noh SJ, Kim KM, Park SH, Hussein UK, Park HS, Park BH, Ha SH, Lee H, Chung MJ (2018). SIRT6 Is Involved in the Progression of Ovarian Carcinomas via beta-Catenin-Mediated Epithelial to Mesenchymal Transition. Front Oncol.

[17] Bai L, Lin G, Sun L, Liu Y, Huang X, Cao C, Guo Y, Xie C (2016). Upregulation of SIRT6 predicts poor prognosis and promotes metastasis of non-small cell lung cancer via the ERK1/2/MMP9 pathway. Oncotarget.

[18] Shen X, Li P, Xu Y, Chen X, Sun H, Zhao Y, Liu M, Zhang W (2017). Association of sirtuins with clinicopathological parameters and overall survival in gastric cancer. Oncotarget.

[19] Yang J, Li Y, Zhang Y, Fang X, Chen N, Zhou X, Wang X (2020). Sirt6 promotes tumorigenesis and drug resistance of diffuse large B-cell lymphoma by mediating PI3K/Akt signaling. J Exp Clin Cancer Res.

[20] Marquardt JU, Fischer K, Baus K, Kashyap A, Ma S, Krupp M, Linke M, Teufel A, Zechner U, Strand D (2013). Sirtuin-6-dependent genetic and epigenetic alterations are associated with poor clinical outcome in hepatocellular carcinoma patients. Hepatology.

[21] Chen A (2011). PARP inhibitors: its role in treatment of cancer. Chin J Cancer.

[22] Cree IA, Charlton P (2017). Molecular chess? Hallmarks of anti-cancer drug resistance. BMC Cancer.

[23] Meehan RS, Chen AP (2016). New treatment option for ovarian cancer: PARP inhibitors. Gynecol Oncol Res Pract.

[24] Carey LA, Sharpless NE (2011). PARP and cancer–if it’s broke, don’t fix it. N Engl J Med.

[25] Tian X, Firsanov D, Zhang Z, Cheng Y, Luo L, Tombline G, Tan R, Simon M, Henderson S, Steffan J (2019). SIRT6 Is Responsible for More Efficient DNA Double-Strand Break Repair in Long-Lived Species. Cell.

[26] Kong Q, Li Y, Liang Q, Xie J, Li X, Fang J (2020). SIRT6-PARP1 is involved in HMGB1 polyADP-ribosylation and acetylation and promotes chemotherapy-induced autophagy in leukemia. Cancer Biol Ther.

[27] Board WCoTE. Soft Tissue and Bone Tumours 5th Edition edn. Lyon (France): International Agency for Research on Cancer; 2020.

[28] Gill J, Ahluwalia MK, Geller D, Gorlick R (2013). New targets and approaches in osteosarcoma. Pharmacol Ther.

[29] Mei Z, Zhang X, Yi J, Huang J, He J, Tao Y (2016). Sirtuins in metabolism, DNA repair and cancer. J Exp Clin Cancer Res.

[30] Yelamos J, Moreno-Lama L, Jimeno J, Ali SO. Immunomodulatory Roles of PARP-1 and PARP-2: Impact on PARP-Centered Cancer Therapies. Cancers (Basel). 2020;12(2). 10.3390/cancers12020392 PMC7072203 32046278

[31] Park HJ, Bae JS, Kim KM, Moon YJ, Park SH, Ha SH, Hussein UK, Zhang Z, Park HS, Park BH (2018). The PARP inhibitor olaparib potentiates the effect of the DNA damaging agent doxorubicin in osteosarcoma. J Exp Clin Cancer Res.

[32] Gao Y, Qu Y, Zhou Q, Ma Y (2019). SIRT6 inhibits proliferation and invasion in osteosarcoma cells by targeting N-cadherin. Oncol Lett.

[33] Xu XZ, Song H, Zhao Y, Zhang L (2020). MiR-654-5p regulated cell progression and tumor growth through targeting SIRT6 in osteosarcoma. Eur Rev Med Pharmacol Sci.

[34] Xue W, Ma L, Wang Z, Zhang W, Zhang X (2019). FOXN3 is downregulated in osteosarcoma and transcriptionally regulates SIRT6, and suppresses migration and invasion in osteosarcoma. Oncol Rep.

[35] Lin H, Hao Y, Zhao Z, Tong Y (2017). Sirtuin 6 contributes to migration and invasion of osteosarcoma cells via the ERK1/2/MMP9 pathway. FEBS Open Bio.

[36] Amin MB, American Joint Committee on Cancer., American Cancer Society. AJCC cancer staging manual, Eight edition / editor-in-chief. In: Amin MB, MD,FCAP; editors, SB Edge. MD, FACS and 16 others ; Donna M. Gress, RHIT, CTR - Technical editor ; Laura R. Meyer, CAPM - Managing editor. edn. Chicago: American Joint Committee on Cancer, Springer; 2017.

[37] Kang MA, Lee J, Ha SH, Lee CM, Kim KM, Jang KY, Park SH. Interleukin4Ralpha (IL4Ralpha) and IL13Ralpha1 Are Associated with the Progress of Renal Cell Carcinoma through Janus Kinase 2 (JAK2)/Forkhead Box O3 (FOXO3) Pathways. Cancers (Basel). 2019;11(9). 10.3390/cancers11091394 PMC6770213 31540495

[38] Kim KM, Hussein UK, Park SH, Kang MA, Moon YJ, Zhang Z, Song Y, Park HS, Bae JS, Park BH (2019). FAM83H is involved in stabilization of beta-catenin and progression of osteosarcomas. J Exp Clin Cancer Res.

[39] Ahn SW, Ahn AR, Ha SH, Hussein UK, Do Yang J, Kim KM, Park HS, Park SH, Yu HC, Jang KY (2020). Expression of FAM83H and ZNF16 are associated with shorter survival of patients with gallbladder carcinoma. Diagn Pathol.

[40] Huang N, Liu Z, Zhu J, Cui Z, Li Y, Yu Y, Sun F, Pan Q, Yang Q (2017). Sirtuin 6 plays an oncogenic role and induces cell autophagy in esophageal cancer cells. Tumour Biol.

[41] Garcia-Peterson LM, Ndiaye MA, Singh CK, Chhabra G, Huang W, Ahmad N (2017). SIRT6 histone deacetylase functions as a potential oncogene in human melanoma. Genes Cancer.

[42] Li Z, Huang J, Shen S, Ding Z, Luo Q, Chen Z, Lu S (2018). SIRT6 drives epithelial-to-mesenchymal transition and metastasis in non-small cell lung cancer via snail-dependent transrepression of KLF4. J Exp Clin Cancer Res.

[43] Bauer I, Grozio A, Lasiglie D, Basile G, Sturla L, Magnone M, Sociali G, Soncini D, Caffa I, Poggi A (2012). The NAD+-dependent histone deacetylase SIRT6 promotes cytokine production and migration in pancreatic cancer cells by regulating Ca2 + responses. J Biol Chem.

[44] Zhang ZG, Qin CY (2014). Sirt6 suppresses hepatocellular carcinoma cell growth via inhibiting the extracellular signalregulated kinase signaling pathway. Mol Med Rep.

[45] Tian K, Chen P, Liu Z, Si S, Zhang Q, Mou Y, Han L, Wang Q, Zhou X (2017). Sirtuin 6 inhibits epithelial to mesenchymal transition during idiopathic pulmonary fibrosis via inactivating TGF-beta1/Smad3 signaling. Oncotarget.

[46] Azuma Y, Yokobori T, Mogi A, Altan B, Yajima T, Kosaka T, Onozato R, Yamaki E, Asao T, Nishiyama M (2015). SIRT6 expression is associated with poor prognosis and chemosensitivity in patients with non-small cell lung cancer. J Surg Oncol.

[47] Park SH, Noh SJ, Kim KM, Bae JS, Kwon KS, Jung SH, Kim JR, Lee H, Chung MJ, Moon WS (2015). Expression of DNA Damage Response Molecules PARP1, gammaH2AX, BRCA1, and BRCA2 Predicts Poor Survival of Breast Carcinoma Patients. Transl Oncol.

[48] Kim KM, Moon YJ, Park SH, Park HJ, Wang SI, Park HS, Lee H, Kwon KS, Moon WS, Lee DG (2016). Individual and Combined Expression of DNA Damage Response Molecules PARP1, gammaH2AX, BRCA1, and BRCA2 Predict Shorter Survival of Soft Tissue Sarcoma Patients. PLoS One.

[49] Cho D, Park H, Park SH, Kim K, Chung M, Moon W, Kang M, Jang K (2015). The expression of DBC1/CCAR2 is associated with poor prognosis of ovarian carcinoma. J Ovarian Res.

[50] Engert F, Kovac M, Baumhoer D, Nathrath M, Fulda S (2017). Osteosarcoma cells with genetic signatures of BRCAness are susceptible to the PARP inhibitor talazoparib alone or in combination with chemotherapeutics. Oncotarget.

[51] Li S, Cui Z, Meng X (2016). Knock-down of PARP-1 Inhibits Proliferation and ERK Signals, Increasing Drug Sensitivity in Osteosarcoma U2OS Cells. Oncol Res.

[52] Lee HJ, Yoon C, Schmidt B, Park DJ, Zhang AY, Erkizan HV, Toretsky JA, Kirsch DG, Yoon SS (2013). Combining PARP-1 inhibition and radiation in Ewing sarcoma results in lethal DNA damage. Mol Cancer Ther.

